# Novel use of a colonic intraprocedural cleansing device for upper gastrointestinal bleeding

**DOI:** 10.1055/a-2210-0055

**Published:** 2023-12-21

**Authors:** Tessa Herman, Morgan Freeman, Nicha Wongjarupong, Vijay Are, Long B. Le, Mohammad Bilal, Brian J. Hanson

**Affiliations:** 1 5635 Department of Medicine, University of Minnesota, Minneapolis, United States; 2 5635 Division of Gastroenterology, Hepatology, and Nutrition, University of Minnesota, Minneapolis, United States; 3 20040 Gastroenterology, Minneapolis VA Medical Center, Minneapolis, United States

A 74-year-old patient with iron deficiency anemia presented for an outpatient upper endoscopy. Two 20–30 mm pedunculated polyps were detected in the gastric body ( Fig. 1 ) and removed with a hot snare. Polypectomy bleeding prompted hemostatic clip placement. However, bleeding persisted, and endoscopic visualization was compromised. The endoscope was exchanged for one with a larger suction channel, but visualization remained inadequate. Therefore, the Pure-Vu EVS System (Motus GI, Tirat Carmel, Israel), a US Food and Drug Administration-approved oversleeve-based intraprocedural cleansing device intended for use in the colon ( Fig. 2 ), was used to improve visualization, allowing further hemostatic interventions.

**Fig. 1:**
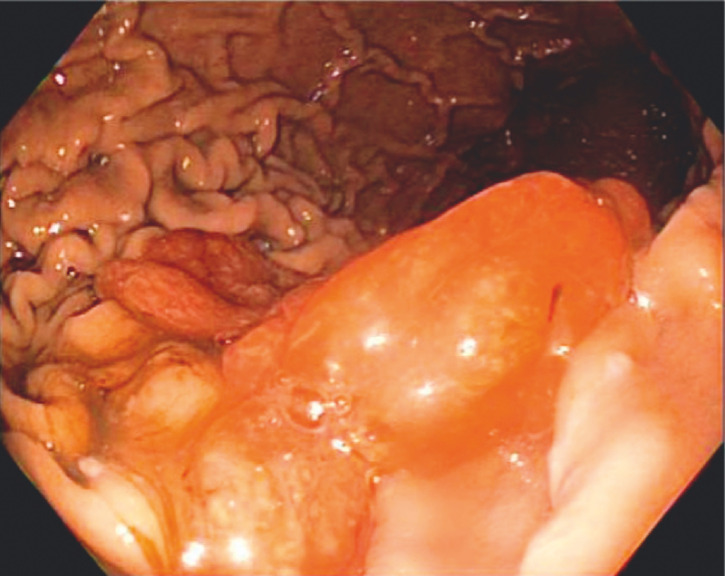
Two 20–30 mm pedunculated polyps were found in the gastric body.

**Fig. 2:**
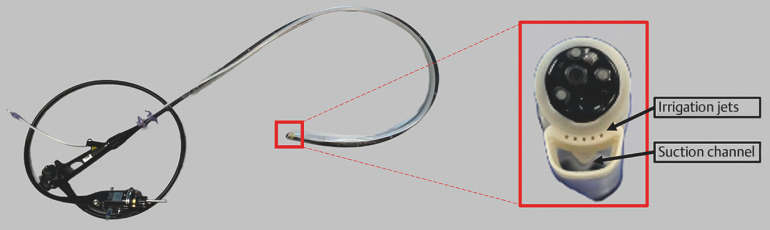
Intraprocedural cleansing device. The over-the-scope device was loaded onto a colonoscope. The device contains five irrigation jets and a large suction channel, and does not inhibit the use of the colonoscope’s working channel.

The endoscope was removed to load the device, which contains five irrigation jets and a large-caliber suction channel attached to a device-specific workstation. The device leaves the working channel free. With the device in place, esophageal intubation was performed without difficulty. The powerful irrigation and suction capabilities of the device allowed rapid clearance of blood and clot ( Fig. 3 , Video 1 ). Additionally, a large clot was suctioned into the device suction channel, and utilizing a manual purge function, the clot was deposited in the gastric antrum. With improved visualization, epinephrine was injected and additional hemostatic clips were placed ( Fig. 4 ). To prevent rebleeding, hemostatic spray was applied ( Fig. 5 ). With the working channel free and endoscope maneuverability uninhibited, the device was left in place throughout the procedure. Ultimately, definitive hemostasis was achieved, and there were no adverse events. Pathology revealed benign foveolar hyperplastic polyps.

**Fig. 3:**
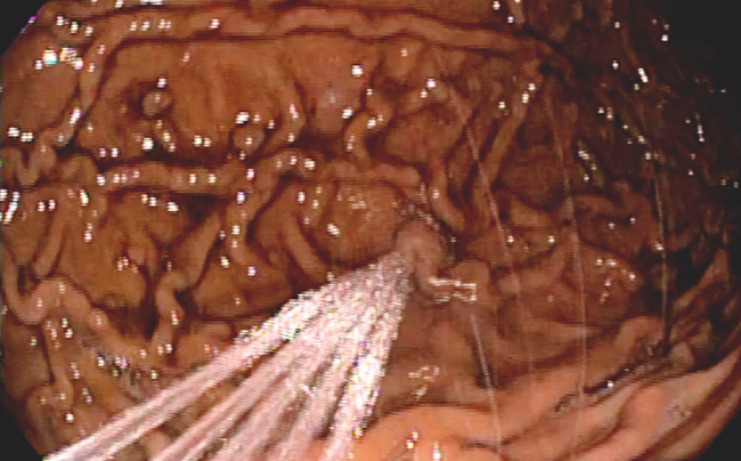
Device irrigation jets. The jets of the intraprocedural cleansing device cleansed the stomach wall.

A colonic intraprocedural cleansing device was used to improve endoscopic visualization during upper gastrointestinal bleeding.Video 1

**Fig. 4:**
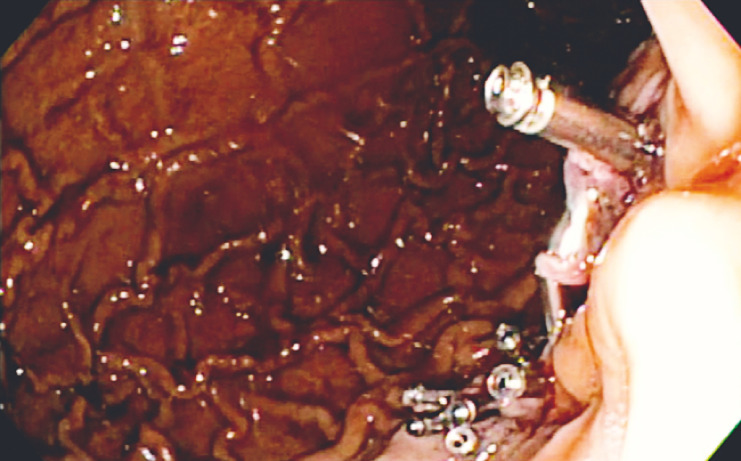
Hemostasis after endoscopic clipping. View of the polypectomy site after multiple hemostatic clips were placed to achieve hemostasis.

**Fig. 5:**
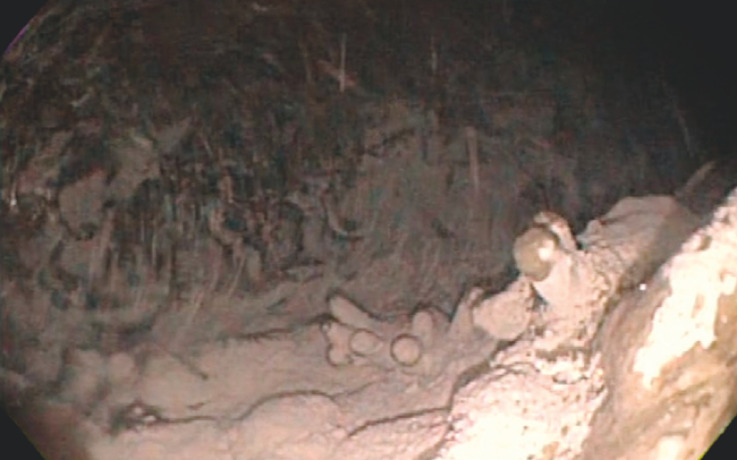
Hemostatic spray application to the polypectomy site. At the end of the procedure, hemostatic spray was applied to the polypectomy site to prevent rebleeding. This was performed easily with the device in place as it does not block the working channel of the endoscope.

While this intraprocedural cleansing system is known to be effective for improving colonic bowel preparation 1 2 3 , this is the first reported case of using this device in the upper gastrointestinal tract. Use of the device was warranted given failure of other conventional methods to improve visualization. The device was an effective method for rapidly improving visualization and allowing for timely hemostatic interventions in the setting of vigorous iatrogenic upper gastrointestinal bleeding.

Endoscopy_UCTN_Code_TTT_1AO_2AD

## Notes

Endoscopy E-Videos https://eref.thieme.de/e-videos
*E-Videos* is an open access online section of the journal *Endoscopy* , reporting on interesting cases and new techniques in gastroenterological endoscopy. All papers include a high-quality video and are published with a Creative Commons CC-BY license. Endoscopy E-Videos qualify for HINARI discounts and waivers and eligibility is automatically checked during the submission process. We grant 100% waivers to articles whose corresponding authors are based in Group A countries and 50% waivers to those who are based in Group B countries as classified by Research4Life (see: https://www.research4life.org/access/eligibility/ ). This section has its own submission website at https://mc.manuscriptcentral.com/e-videos .
